# Supplementary material for: OrthoFinder: phylogenetic orthology inference for comparative genomics
Source: Genome Biol. 2019 Nov 14;20:238. doi: 10.1186/s13059-019-1832-y (PMC6857279; doi:10.1186/s13059-019-1832-y)
Supplement: Supplementary file 1 — Additional file 1. Supplementary figures and tables. [file 13059_2019_1832_MOESM1_ESM.pdf]

Supplementary Figures

Figure S1

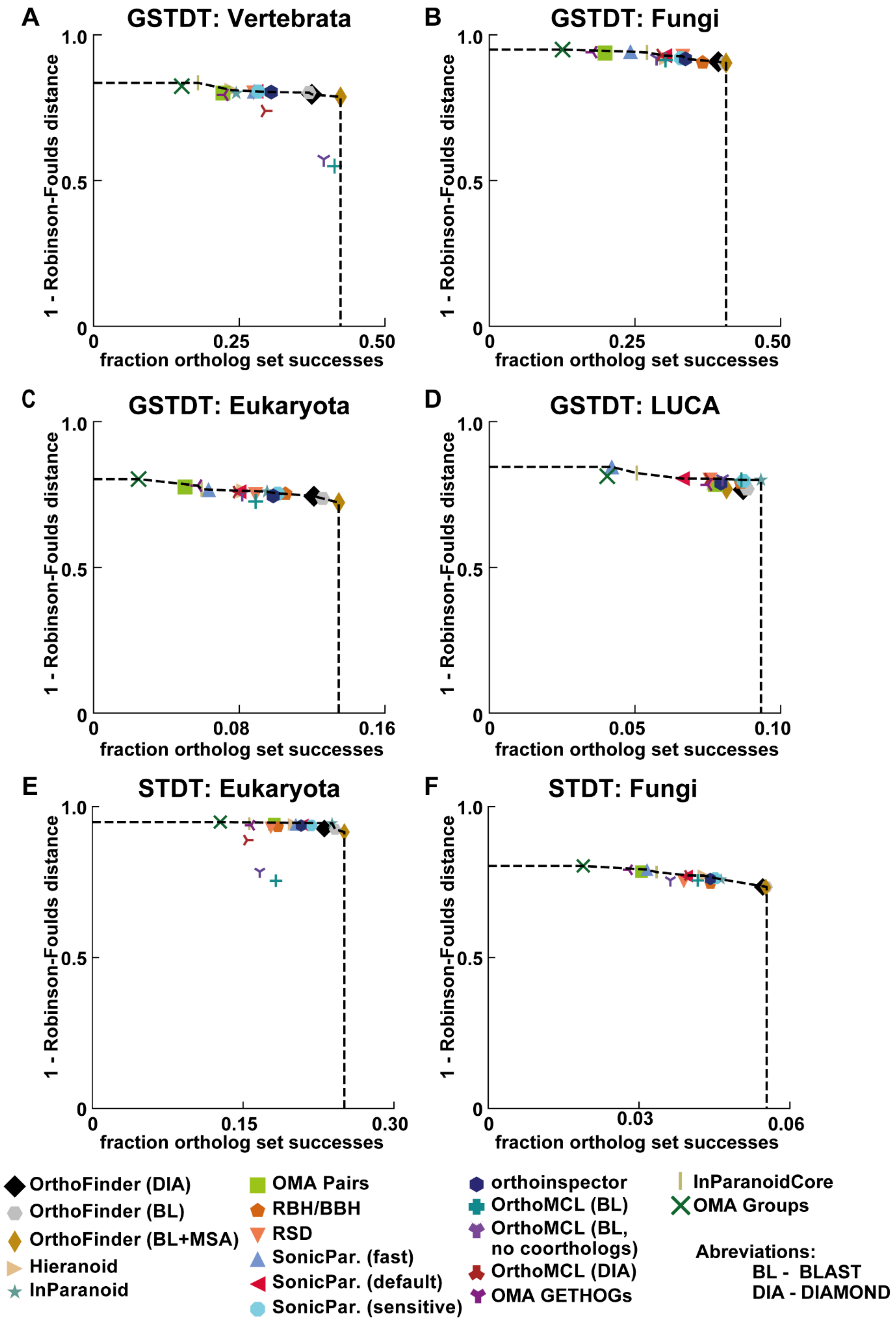

Figure S2

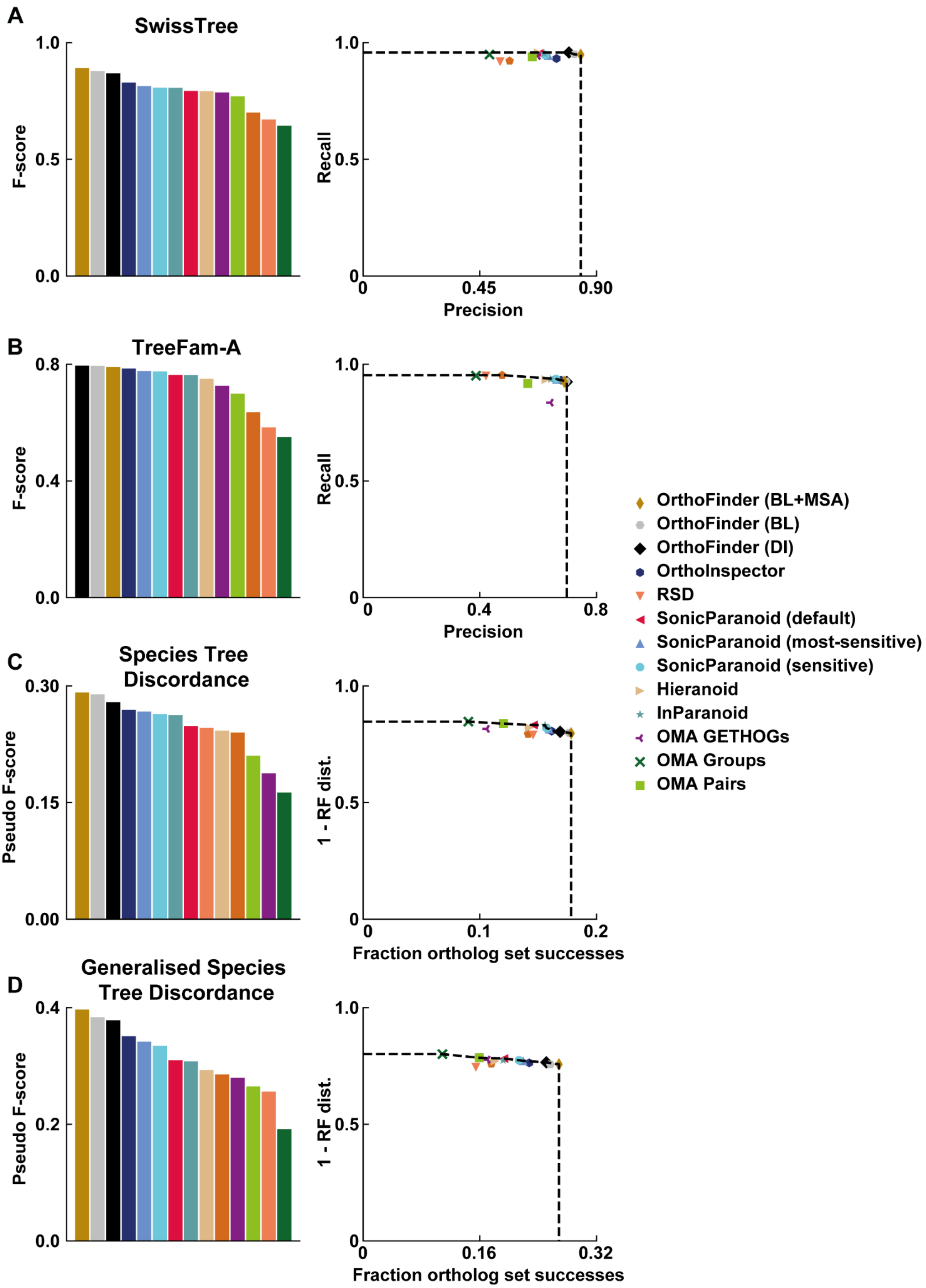
A
SwissTree
F-score
1.0
0.5
0.0
Recall
1.0
0.5
0
0.45
0.90
Precision
B
TreeFam-A
F-score
0.8
0.4
0.0
Recall
1.0
0.5
0
0.4
0.8
Precision
C
Species Tree Discordance
Pseudo F-score
0.30
0.15
0.00
1 - RF dist.
1.0
0.5
0
0.1
0.2
Fraction ortholog set successes
D
Generalised Species Tree Discordance
Pseudo F-score
0.4
0.2
0.0
1 - RF dist.
1.0
0.5
0
0.16
0.32
Fraction ortholog set successes
OrthoFinder (BL+MSA)
OrthoFinder (BL)
OrthoFinder (DI)
OrthoInspector
RSD
SonicParanoid (default)
SonicParanoid (most-sensitive)
SonicParanoid (sensitive)
Hieranoid
InParanoid
OMA GETHOGs
OMA Groups
OMA Pairs

Figure S3

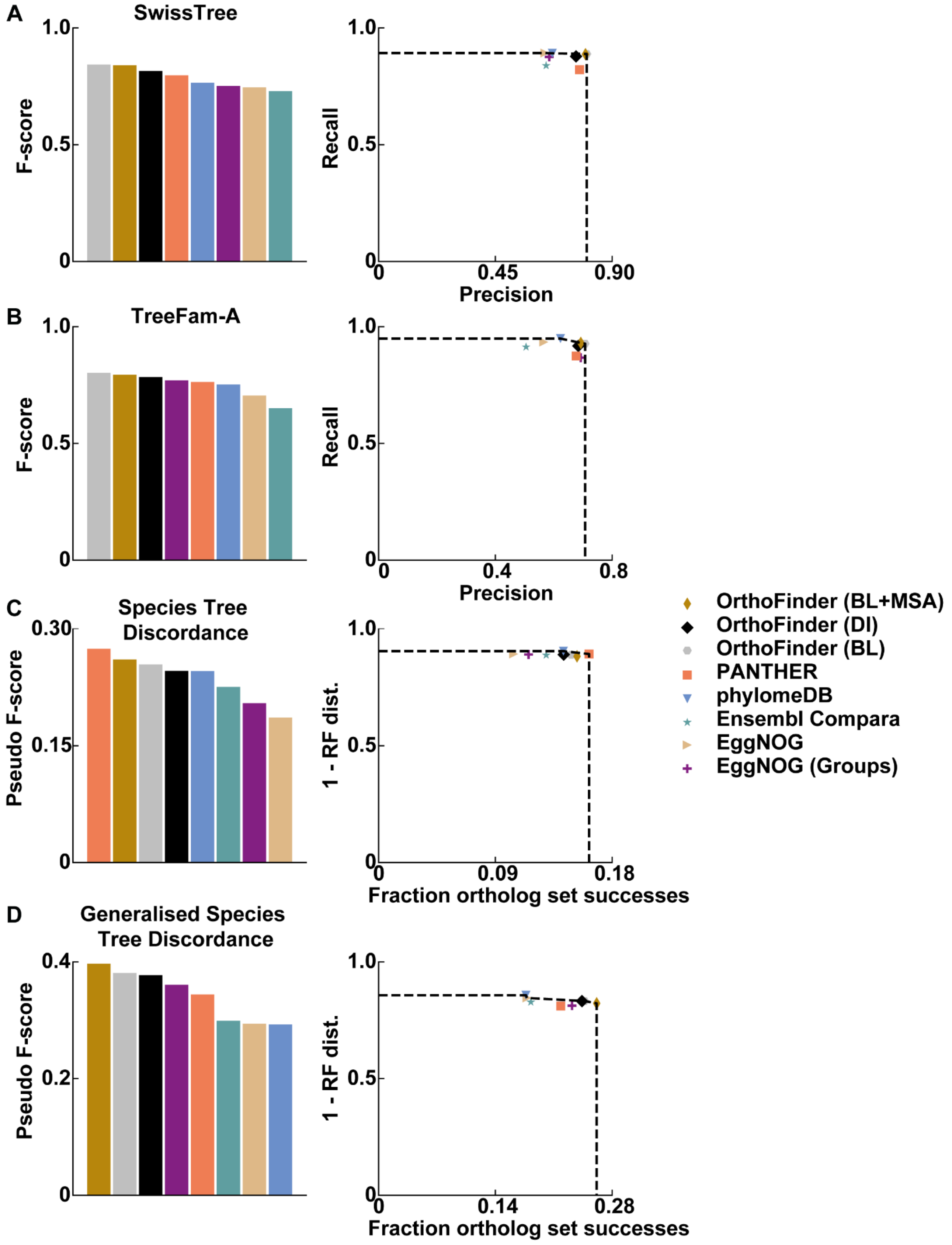
A
SwissTree
F-score
1.0
0.5
0
Recall
0.45
0.90
Precision
B
TreeFam-A
0.4
0.8
C
Species Tree Discordance
Pseudo F-score
0.30
0.15
1 - RF dist.
0.09
0.18
Fraction ortholog set successes
D
Generalised Species Tree Discordance
0.4
0.2
0.14
0.28
OrthoFinder (BL+MSA)
OrthoFinder (DI)
OrthoFinder (BL)
PANTHER
phylomeDB
Ensembl Compara
EggNOG
EggNOG (Groups)

Figure S4

A. **Primary Overlap depth**

B. **Archetype** **Sub-overlaps** **Constraints**

Case A d=(1,0)

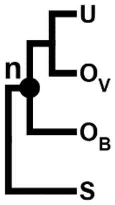

sub-cases

	1	2	3	4
S&U	F	T	F	T
S &O	F	F	T	T

Constraints: ¬U&O

Case B d=(1,1)

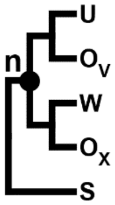

sub-cases

	1	2	3	4	5	6	7	8
S&O	F	F	F	F	T	T	T	T
S&U	F	T	F	T	F	T	F	T
S&W	F	F	T	T	F	T	F	T

Constraints: ¬U&O, ¬W&O, ¬U &W

Case C d=(2,0)

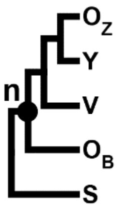

sub-cases

	1	2	3	4	5	6	7	8	9	10	11	12	13	14	15	16
S&V	F	F	F	F	F	F	F	F	T	T	T	T	T	T	T	T
S&Y	F	F	F	F	T	T	T	T	F	F	F	F	T	T	T	T
S&O	F	F	T	T	F	F	T	T	F	F	T	T	F	F	T	T
V&Y	F	T	F	T	F	T	F	T	F	T	F	T	F	T	F	T

Constraints: ¬Y&O, ¬V&O

C.

Case	Input tree	DLC before	Reconciled tree	DLC after
-	$((O_A, O_B), S)$	1D = 1	$((O_A, O_B), S)$	1D = 1
A.1	$((U, O_V), O_B), S)$	D + L = 2	$(((O_V, O_B), U), S)$	D + C = 1.5
A.2	$((U, O_V), O_B), S)$	2D + 2L = 4	$((U, O_V), (O_B, S))$	D + C = 1.5
A.3	$((U, O_V), O_B), S)$	2D + 2L = 4	$(((O_V, O_B), U), S)$	2D + L + C = 3.5
A.4	$((U, O_V), O_B), S)$	2D + 2L = 4	$(((O_V, O_B), U), S)$	2D + L + C = 3.5
B.1	$((U, O_V), (W, O_X)), S)$	D + 2L = 3	$((W, (U(O_V, O_X))), S)$	D + 2C = 2
B.2	$((U, O_V), (W, O_X)), S)$	2D + 3L = 5	$((U, O_V), (S, O_X)), W)$	D + 2C = 2
B.3	$((U, O_V), (W, O_X)), S)$	2D + 3L = 5	$((W, O_X), (S, O_V)), U)$	D + 2C = 2
B.4	$((U, O_V), (W, O_X)), S)$	2D + L = 3	$((U, O_V), (O_X, (W, S)))$	D + 2C = 2
B.5	$((U, O_V), (W, O_X)), S)$	2D + 3L = 3	$((U, O_V), (W, O_X)), S)$	2D + 3L = 3
B.6	$((U, O_V), (W, O_X)), S)$	2D + 3L = 5	$((O_X, O_V), (W, U)), S)$	2D + L + 2C = 4
B.7	$((U, O_V), (W, O_X)), S)$	2D + 3L = 5	$((O_X, O_V), (W, U)), S)$	2D + L + 2C = 4
B.8	$((U, O_V), (W, O_X)), S)$	2D + 2L = 4	$((O_X, O_V), (W, U)), S)$	2D + 2C = 4
C.1	$((((O_Z, Y), V), O_B), S)$	D + 2L = 3	$((((O_Z, O_B), Y), V), S)$	D + 2C = 2
C.2	$((((O_Z, Y), V), O_B), S)$	2D + 2L = 4	$(((O_Z, Y), (V, O_B)), S)$	D + C = 1.5
C.3	$((((O_Z, Y), V), O_B), S)$	3D + 4L = 7	$((((O_Z, O_B), Y), V), S)$	2D + 2L + 2C = 5
C.4	$((((O_Z, Y), V), O_B), S)$	3D + 3L = 6	$(((O_Z, Y), (V, O_B)), S)$	2D + L + C = 3.5
C.5	$((((O_Z, Y), V), O_B), S)$	2D + 4L = 6	$(((O_Z, Y), V), (O_B, S))$	D + L + C = 2.5
C.6	$((((O_Z, Y), V), O_B), S)$	2D + 4L = 6	$(((O_Z, Y), (V, O_B)), S)$	2D + L + C = 3.5
C.7	$((((O_Z, Y), V), O_B), S)$	3D + 2L = 5	$(((O_Z, Y), V), (O_B, S))$	3D + L + C = 4.5
C.8	$((((O_Z, Y), V), O_B), S)$	3D + 2L = 5	$(((O_Z, Y), (V, O_B)), S)$	2D + C = 2.5
C.9	$((((O_Z, Y), V), O_B), S)$	2D + 3L = 5	$(((O_Z, Y), V), (O_B, S))$	D + L + C = 2.5
C.10	$((((O_Z, Y), V), O_B), S)$	3D + 3L = 6	$(((O_Z, Y), V), (O_B, S))$	2D + L + C = 3.5
C.11	$((((O_Z, Y), V), O_B), S)$	3D + 3L = 6	$(((O_Z, Y), V), (O_B, S))$	3D + 2L + C = 5.5
C.12	$((((O_Z, Y), V), O_B), S)$	3D + 2L = 5	$(((O_Z, Y), (V, O_B)), S)$	2D + C = 2.5
C.13	$((((O_Z, Y), V), O_B), S)$	3D + 3L = 6	$(((O_Z, Y), (V, O_B)), S)$	2D + L + C = 3.5
C.14	$((((O_Z, Y), V), O_B), S)$	3D + 3L = 6	$(((O_Z, Y), (V, O_B)), S)$	2D + L + C = 3.5
C.15	$((((O_Z, Y), V), O_B), S)$	2D + 2L = 4	$((((O_Z, Y), V), O_B), S)$	2D + 2L = 4
C.16	$((((O_Z, Y), V), O_B), S)$	3D + 2L = 5	$(((O_Z, Y), (V, O_B)), S)$	2D + C = 2.5

Supplementary Figure Legends

Figure S1

Results of the individual Generalised Species Tree Discordance tests (GSTDT) and Species Tree Discordance Tests (STDT) from the Quest for Orthologs benchmarks for four clades of species. A) GSTDT Vertebrata B) GSTDT Fungi C) GSTDT Eukaryota D) GSTDT LUCA (Last Universal Common Ancestor, a species set covering bacteria, archaea and eukaryotes) E) STDT Eukaryota F) STDT Fungi. See methods for description of Quest for Orthologs benchmarks.

Figure S2

The 2018 Quest for Orthologs benchmark results for OrthoFinder versus software tools. A-B) F-score versus orthologs from gold standard trees C-D) Pseudo F-score on species tree discordance test E-F) Precision & Recall versus gold standard trees G-H) 1 minus normalised Robinson-Foulds (RF) distance & fraction of ortholog set successes on species tree discordance tests.

Figure S3

The 2011 Quest for Orthologs benchmark results for OrthoFinder versus online databases. A-B) F-score versus orthologs from gold standard trees C-D) Pseudo F-score on species tree discordance test E-F) Precision & Recall versus gold standard trees G-H) 1 minus normalised Robinson-Foulds (RF) distance & fraction of ortholog set successes on species tree discordance tests.

Figure S4

Deterministic tree reconciliation applied to nodes within a gene tree. If the sets of species below a node, n, overlap the node is analysed to find if there is a more parsimonious interpretation of the subtree under the duplication, loss, deep-coalescence (DLC) model. The analysis is done in the context of the sister clade, S, and the descendant clades, U, V, W, X, Y, Z and O. These clades may contain single or multiple genes. The overlapping clades under the two descendants of n are identified, down to a total combined depth of 2 (so that the problem remains tractable). Each case has a number of possible sub-cases according to whether the species sets in the clades overlap. The notation X&Y means that the species set for the genes in clade X overlap with the species sets

in the clade Y. In order for a node to fit an archetype (the overlap in n’s descendants is in the clades ‘O’), constraints arise on some of the sub-overlaps. T=True, F=False.

Table S1a: OrthoFinder & Software Methods Accuracy

	SwissTree			TreeFam-A			STDT			GSTDT			
	R	P	F	R	P	F	Olog. recov	1-RF	Psu. F	Olog. recov	1-RF	Psu. F	Ave (psu.) F
OrthoFinder	0.761	0.878	0.816	0.684	0.918	0.784	0.143	0.890	0.246	0.244	0.832	0.377	0.556
OrthoFinder (BL)	0.802	0.889	0.843	0.707	0.927	0.803	0.148	0.890	0.254	0.247	0.832	0.381	0.570
OrthoFinder (BL+MSA)	0.797	0.888	0.840	0.693	0.931	0.794	0.153	0.883	0.261	0.262	0.822	0.397	0.573
Hieranoid	0.691	0.883	0.775	0.604	0.926	0.731	0.122	0.912	0.215	0.175	0.850	0.291	0.503
InParanoid	0.688	0.883	0.774	0.632	0.929	0.752	0.142	0.915	0.246	0.191	0.848	0.311	0.521
OMA Pairs	0.652	0.883	0.751	0.578	0.903	0.705	0.106	0.920	0.190	0.137	0.845	0.236	0.470
RBH/BBH	0.621	0.884	0.730	0.493	0.948	0.648	0.114	0.896	0.203	0.209	0.840	0.335	0.479
RSD	0.572	0.884	0.695	0.452	0.949	0.612	0.108	0.898	0.193	0.193	0.849	0.314	0.453
SonicParanoid (fast)	0.656	0.888	0.754	0.591	0.927	0.722	0.117	0.921	0.208	0.156	0.858	0.264	0.487
SonicParanoid (default)	0.688	0.892	0.777	0.625	0.925	0.746	0.124	0.913	0.219	0.181	0.851	0.299	0.510
SonicParanoid (sensitive)	0.716	0.891	0.794	0.641	0.926	0.758	0.131	0.908	0.230	0.200	0.845	0.323	0.526
orthoinspector	0.701	0.877	0.779	0.655	0.927	0.768	0.126	0.907	0.221	0.205	0.842	0.330	0.524
OrthoMCL (BL)	0.652	0.834	0.732	0.594	0.846	0.698	0.112	0.755	0.195	0.223	0.715	0.340	0.491
OrthoMCL (no coorths)	0.611	0.829	0.703	0.505	0.854	0.635	0.101	0.780	0.180	0.211	0.730	0.327	0.461
OrthoMCL (DI)	0.591	0.884	0.708	0.519	0.924	0.664	0.097	0.865	0.174	0.186	0.824	0.303	0.462
OMA Groups	0.476	0.881	0.618	0.390	0.946	0.552	0.073	0.930	0.136	0.086	0.867	0.156	0.365
InParanoidCore	0.530	0.884	0.663	0.476	0.952	0.635	0.095	0.916	0.172	0.140	0.877	0.241	0.428
OMA GETHOGs	0.661	0.885	0.756	0.610	0.836	0.705	0.093	0.916	0.169	0.134	0.840	0.231	0.465

Table S1b: OrthoFinder (default) v other software

	SwissTree			TreeFam-A			STDT			GSTDT			Ave (psu.) F
	R	P	F	R	P	F	Olog. recov	1-RF	Psu. F	Olog. recov	1-RF	Psu. F	
OrthoFinder	0.0%	0.0%	0.0%	0.0%	0.0%	0.0%	0.0%	0.0%	0.0%	0.0%	0.0%	0.0%	0.0%
OrthoFinder (BL)	-5.1%	-1.2%	-3.3%	-3.3%	-1.0%	-2.3%	-3.9%	0.1%	-3.3%	-1.3%	-0.1%	-1.0%	-2.6%
OrthoFinder (BL+MSA)	-4.5%	-1.0%	-2.9%	-1.2%	-1.4%	-1.3%	-6.7%	0.8%	-5.7%	-6.7%	1.1%	-5.0%	-3.0%
Hieranoid	9.2%	-0.5%	4.9%	11.8%	-0.8%	6.8%	14.6%	-2.3%	12.6%	28.1%	-2.2%	22.9%	9.5%
InParanoid	9.6%	-0.5%	5.1%	7.7%	-1.2%	4.1%	0.2%	-2.7%	-0.2%	21.8%	-1.9%	17.4%	6.3%
OMA Pairs	14.3%	-0.6%	8.0%	15.5%	1.6%	10.1%	25.9%	-3.2%	22.9%	43.8%	-1.6%	37.4%	15.4%
RBH/BBH	18.4%	-0.7%	10.5%	28.0%	-3.2%	17.3%	19.8%	-0.7%	17.5%	14.2%	-0.9%	11.2%	13.8%
RSD	24.9%	-0.7%	14.8%	34.0%	-3.3%	22.0%	24.1%	-0.8%	21.5%	20.9%	-2.0%	16.7%	18.4%
SonicParanoid (fast)	13.8%	-1.1%	7.5%	13.7%	-1.0%	7.9%	18.0%	-3.4%	15.6%	36.1%	-3.0%	30.1%	12.4%
SonicParanoid (default)	9.6%	-1.5%	4.7%	8.6%	-0.8%	4.8%	13.0%	-2.4%	11.1%	25.6%	-2.3%	20.7%	8.2%
SonicParanoid (sensitive)	5.9%	-1.4%	2.7%	6.3%	-0.9%	3.4%	7.9%	-1.9%	6.6%	18.0%	-1.5%	14.3%	5.3%
orthoinspector	8.0%	0.2%	4.5%	4.3%	-1.0%	2.1%	11.8%	-1.8%	10.1%	16.0%	-1.2%	12.6%	5.7%
OrthoMCL (BL)	14.4%	5.1%	10.3%	13.2%	7.8%	11.0%	21.4%	15.3%	20.6%	8.6%	14.0%	9.9%	11.6%
OrthoMCL (no coorths)	19.8%	5.6%	13.7%	26.1%	7.0%	19.0%	28.9%	12.4%	27.0%	13.5%	12.2%	13.2%	17.0%
OrthoMCL (DI)	22.4%	-0.6%	13.2%	24.2%	-0.6%	15.3%	32.1%	2.8%	29.2%	23.9%	1.0%	19.7%	16.8%
OMA Groups	37.5%	-0.3%	24.3%	43.1%	-3.0%	29.6%	48.7%	-4.3%	44.9%	64.9%	-4.1%	58.7%	34.3%
InParanoidCore	30.3%	-0.7%	18.7%	30.4%	-3.6%	19.0%	33.6%	-2.8%	30.1%	42.6%	-5.2%	36.0%	23.0%
OMA GETHOGs	13.2%	-0.7%	7.3%	10.9%	9.0%	10.1%	34.8%	-2.8%	31.3%	45.2%	-0.9%	38.9%	16.3%
OF v best non-OF	5.9%	-1.5%	2.7%	4.3%	-3.6%	2.1%	0.2%	-4.3%	-0.2%	8.6%	-5.2%	9.9%	5.3%
OF v worst non-OF	37.5%	5.6%	24.3%	43.1%	9.0%	29.6%	48.7%	15.3%	44.9%	64.9%	14.0%	58.7%	34.3%

Table S1c: OrthoFinder (BLAST) v other software

	SwissTree			TreeFam-A			STDT			GSTDT			Ave (psu.) F
	R	P	F	R	P	F	Olog. recov	1-RF	Psu. F	Olog. recov	1-RF	Psu. F	
OrthoFinder	5.1%	1.2%	3.3%	3.3%	1.0%	2.3%	3.9%	-0.1%	3.3%	1.3%	0.1%	1.0%	2.6%
OrthoFinder (BL)	0.0%	0.0%	0.0%	0.0%	0.0%	0.0%	0.0%	0.0%	0.0%	0.0%	0.0%	0.0%	0.0%
OrthoFinder (BL+MSA)	0.6%	0.1%	0.4%	2.1%	-0.3%	1.0%	-3.0%	0.7%	-2.4%	-5.5%	1.2%	-4.0%	-0.5%
Hieranoid	13.8%	0.7%	8.1%	14.7%	0.2%	9.0%	17.9%	-2.4%	15.5%	29.1%	-2.1%	23.7%	11.8%
InParanoid	14.2%	0.7%	8.3%	10.7%	-0.1%	6.3%	4.1%	-2.8%	3.1%	22.8%	-1.9%	18.3%	8.7%
OMA Pairs	18.7%	0.6%	11.0%	18.3%	2.6%	12.2%	28.8%	-3.3%	25.4%	44.5%	-1.6%	38.1%	17.5%
RBH/BBH	22.5%	0.5%	13.5%	30.4%	-2.2%	19.2%	22.9%	-0.8%	20.2%	15.3%	-0.9%	12.1%	16.0%
RSD	28.7%	0.5%	17.6%	36.2%	-2.3%	23.8%	27.1%	-0.9%	24.1%	21.9%	-2.0%	17.5%	20.5%
SonicParanoid (fast)	18.3%	0.1%	10.5%	16.5%	0.0%	10.1%	21.2%	-3.5%	18.4%	37.0%	-3.0%	30.8%	14.6%
SonicParanoid (default)	14.2%	-0.3%	7.9%	11.6%	0.3%	7.0%	16.3%	-2.5%	14.1%	26.5%	-2.3%	21.5%	10.5%
SonicParanoid (sensitive)	10.8%	-0.2%	5.9%	9.4%	0.2%	5.6%	11.4%	-2.0%	9.7%	19.1%	-1.5%	15.1%	7.8%
orthoinspector	12.7%	1.4%	7.7%	7.4%	0.1%	4.4%	15.2%	-1.9%	13.1%	17.1%	-1.1%	13.5%	8.1%
OrthoMCL (BL)	18.8%	6.2%	13.3%	16.0%	8.8%	13.0%	24.4%	15.2%	23.2%	9.8%	14.1%	10.8%	13.9%
OrthoMCL (no coorths)	23.9%	6.7%	16.6%	28.6%	7.9%	20.9%	31.7%	12.3%	29.4%	14.6%	12.3%	14.1%	19.1%
OrthoMCL (DI)	26.3%	0.6%	16.0%	26.7%	0.4%	17.2%	34.8%	2.7%	31.5%	24.8%	1.1%	20.5%	18.9%
OMA Groups	40.7%	0.9%	26.8%	44.9%	-2.0%	31.2%	50.7%	-4.4%	46.7%	65.4%	-4.0%	59.1%	36.0%
InParanoidCore	33.9%	0.5%	21.4%	32.7%	-2.6%	20.9%	36.1%	-2.9%	32.5%	43.4%	-5.1%	36.7%	25.0%
OMA GETHOGs	17.7%	0.5%	10.3%	13.8%	9.9%	12.2%	37.3%	-2.9%	33.6%	45.9%	-0.9%	39.5%	18.4%
OF v best non-OF	10.8%	-0.3%	5.9%	7.4%	-2.6%	4.4%	4.1%	-4.4%	3.1%	9.8%	-5.1%	10.8%	7.8%
OF v worst non-OF	40.7%	6.7%	26.8%	44.9%	9.9%	31.2%	50.7%	15.2%	46.7%	65.4%	14.1%	59.1%	36.0%

Table S1d: OrthoFinder (BLAST+MSA) v other software

	SwissTree			TreeFam-A			STDT			GSTDT			Ave (psu.) F
	R	P	F	R	P	F	Olog. recov	1-RF	Psu. F	Olog. recov	1-RF	Psu. F	
OrthoFinder	4.5%	1.0%	2.9%	1.2%	1.4%	1.3%	6.7%	-0.8%	5.7%	6.7%	-1.1%	5.0%	3.0%
OrthoFinder (BL)	-0.6%	-0.1%	-0.4%	-2.1%	0.3%	-1.0%	3.0%	-0.7%	2.4%	5.5%	-1.2%	4.0%	0.5%
OrthoFinder (BL+MSA)	0.0%	0.0%	0.0%	0.0%	0.0%	0.0%	0.0%	0.0%	0.0%	0.0%	0.0%	0.0%	0.0%
Hieranoid	13.3%	0.5%	7.7%	12.9%	0.5%	8.0%	20.4%	-3.1%	17.6%	33.0%	-3.3%	26.8%	12.2%
InParanoid	13.7%	0.5%	7.9%	8.8%	0.2%	5.3%	6.9%	-3.5%	5.5%	27.1%	-3.0%	21.5%	9.1%
OMA Pairs	18.2%	0.5%	10.7%	16.6%	3.0%	11.3%	30.9%	-4.0%	27.2%	47.6%	-2.7%	40.5%	17.9%
RBH/BBH	22.1%	0.4%	13.1%	28.9%	-1.8%	18.4%	25.2%	-1.5%	22.2%	20.0%	-2.0%	15.6%	16.4%
RSD	28.3%	0.4%	17.3%	34.8%	-1.9%	23.0%	29.2%	-1.7%	25.9%	26.3%	-3.1%	20.8%	20.9%
SonicParanoid (fast)	17.8%	0.0%	10.2%	14.7%	0.3%	9.1%	23.5%	-4.2%	20.3%	40.4%	-4.1%	33.6%	15.0%
SonicParanoid (default)	13.7%	-0.4%	7.5%	9.7%	0.6%	6.1%	18.8%	-3.3%	16.2%	30.6%	-3.4%	24.6%	11.0%
SonicParanoid (sensitive)	10.2%	-0.4%	5.5%	7.5%	0.5%	4.6%	14.0%	-2.8%	11.9%	23.6%	-2.7%	18.5%	8.2%
orthoinspector	12.1%	1.2%	7.3%	5.4%	0.4%	3.4%	17.7%	-2.6%	15.2%	21.7%	-2.3%	17.0%	8.5%
OrthoMCL (BL)	18.3%	6.0%	12.9%	14.3%	9.1%	12.1%	26.7%	14.5%	25.1%	14.8%	13.1%	14.4%	14.3%
OrthoMCL (no coorths)	23.4%	6.6%	16.3%	27.0%	8.2%	20.1%	33.7%	11.7%	31.2%	19.4%	11.2%	17.5%	19.5%
OrthoMCL (DI)	25.9%	0.5%	15.7%	25.1%	0.7%	16.4%	36.7%	2.0%	33.2%	29.0%	-0.1%	23.6%	19.3%
OMA Groups	40.4%	0.8%	26.5%	43.8%	-1.6%	30.5%	52.2%	-5.1%	48.0%	67.3%	-5.1%	60.7%	36.3%
InParanoidCore	33.5%	0.4%	21.1%	31.2%	-2.3%	20.0%	38.0%	-3.6%	34.1%	46.5%	-6.3%	39.2%	25.3%
OMA GETHOGs	17.1%	0.3%	10.0%	12.0%	10.2%	11.3%	39.2%	-3.6%	35.2%	48.9%	-2.1%	41.9%	18.8%
OF v best non-OF	10.2%	-0.4%	5.5%	5.4%	-2.3%	3.4%	6.9%	-5.1%	5.5%	14.8%	-6.3%	14.4%	8.2%
OF v worst non-OF	40.4%	6.6%	26.5%	43.8%	10.2%	30.5%	52.2%	14.5%	48.0%	67.3%	13.1%	60.7%	36.3%

Table S1e: OrthoFinder & Ortholog Database Accuracy

	SwissTree			TreeFam-A			STDT			GSTDT			
	R	P	F	R	P	F	Olog. recov	1-RF	Psu. F	Olog. recov	1-RF	Psu. F	Ave (psu.) F
OrthoFinder	0.761	0.878	0.816	0.684	0.918	0.784	0.143	0.890	0.246	0.244	0.832	0.377	0.556
OrthoFinder (BL)	0.802	0.889	0.843	0.707	0.927	0.803	0.148	0.890	0.254	0.247	0.832	0.381	0.570
OrthoFinder (BL+MSA)	0.797	0.888	0.840	0.693	0.931	0.794	0.153	0.883	0.261	0.262	0.822	0.397	0.573
EggNOG	0.641	0.893	0.746	0.566	0.934	0.705	0.104	0.893	0.186	0.178	0.845	0.294	0.483
Ensembl Compara	0.646	0.839	0.730	0.505	0.913	0.651	0.129	0.888	0.226	0.183	0.828	0.299	0.476
PANTHER 8.0 (all)	0.775	0.821	0.797	0.677	0.874	0.763	0.162	0.893	0.275	0.218	0.811	0.344	0.545
phylomeDB	0.670	0.892	0.765	0.623	0.949	0.752	0.142	0.905	0.246	0.177	0.857	0.293	0.514

Table S1f: OrthoFinder (default) v other

	SwissTree			TreeFam-A			STDT			GSTDT			
	R	P	F	R	P	F	Olog. recov	1-RF	Psu. F	Olog. recov	1-RF	Psu. F	Ave (psu.) F
OrthoFinder	0.0%	0.0%	0.0%	0.0%	0.0%	0.0%	0.0%	0.0%	0.0%	0.0%	0.0%	0.0%	0.0%
OrthoFinder (BL)	-5.1%	-1.2%	-3.3%	-3.3%	-1.0%	-2.3%	-3.9%	0.1%	-3.3%	-1.3%	-0.1%	-1.0%	-2.6%
OrthoFinder (BL+MSA)	-4.5%	-1.0%	-2.9%	-1.2%	-1.4%	-1.3%	-6.7%	0.8%	-5.7%	-6.7%	1.1%	-5.0%	-3.0%
EggNOG	15.8%	-1.6%	8.5%	17.3%	-1.8%	10.1%	27.1%	-0.3%	24.2%	27.1%	-1.6%	22.1%	13.1%
Ensembl Compara	15.2%	4.5%	10.5%	26.2%	0.5%	17.0%	9.5%	0.3%	8.3%	25.2%	0.5%	20.7%	14.3%
PANTHER 8.0 (all)	-1.7%	6.5%	2.2%	1.1%	4.7%	2.7%	-12.0%	-0.3%	-10.4%	10.4%	2.5%	8.7%	2.0%
phylomeDB	12.0%	-1.5%	6.2%	8.9%	-3.3%	4.0%	0.2%	-1.6%	-0.1%	27.6%	-3.0%	22.3%	7.5%
**OF v best non-OF**	-1.7%	-1.6%	2.2%	1.1%	-3.3%	2.7%	-12.0%	-1.6%	-10.4%	10.4%	-3.0%	8.7%	2.0%
**OF v worst non-OF**	15.8%	6.5%	10.5%	26.2%	4.7%	17.0%	27.1%	0.3%	24.2%	27.6%	2.5%	22.3%	14.3%

Table S1g: OrthoFinder (BLAST) v other

	SwissTree			TreeFam-A			STDT			GSTDT			
	R	P	F	R	P	F	Olog. recov	1-RF	Psu. F	Olog. recov	1-RF	Psu. F	Ave. (psu.) F
OrthoFinder	5.1%	1.2%	3.3%	3.3%	1.0%	2.3%	3.9%	-0.1%	3.3%	1.3%	0.1%	1.0%	2.6%
OrthoFinder (BL)	0.0%	0.0%	0.0%	0.0%	0.0%	0.0%	0.0%	0.0%	0.0%	0.0%	0.0%	0.0%	0.0%
OrthoFinder (BL+MSA)	0.6%	0.1%	0.4%	2.1%	-0.3%	1.0%	-3.0%	0.7%	-2.4%	-5.5%	1.2%	-4.0%	-0.5%
EggNOG	20.1%	-0.4%	11.5%	20.0%	-0.7%	12.2%	29.9%	-0.4%	26.8%	28.0%	-1.6%	22.9%	15.4%
Ensembl Compara	19.5%	5.6%	13.5%	28.6%	1.5%	18.9%	13.0%	0.1%	11.3%	26.1%	0.5%	21.5%	16.5%
PANTHER 8.0 (all)	3.4%	7.6%	5.5%	4.4%	5.7%	5.0%	-8.5%	-0.4%	-7.4%	11.6%	2.6%	9.7%	4.5%
phylomeDB	16.5%	-0.3%	9.3%	11.9%	-2.3%	6.3%	4.0%	-1.7%	3.2%	28.5%	-2.9%	23.1%	9.9%
**OF v best non-OF**	3.4%	-0.4%	5.5%	4.4%	-2.3%	5.0%	-8.5%	-1.7%	-7.4%	11.6%	-2.9%	9.7%	4.5%
**OF v worst non-OF**	20.1%	7.6%	13.5%	28.6%	5.7%	18.9%	29.9%	0.1%	26.8%	28.5%	2.6%	23.1%	16.5%

Table S1h: OrthoFinder (BLAST + MSA) v other

	SwissTree			TreeFam-A			STDT			GSTDT			
	R	P	F	R	P	F	Olog. recov	1-RF	Psu. F	Olog. recov	1-RF	Psu. F	Ave. (psu.) F
OrthoFinder	4.5%	1.0%	2.9%	1.2%	1.4%	1.3%	6.7%	-0.8%	5.7%	6.7%	-1.1%	5.0%	3.0%
OrthoFinder (BL)	-0.6%	-0.1%	-0.4%	-2.1%	0.3%	-1.0%	3.0%	-0.7%	2.4%	5.5%	-1.2%	4.0%	0.5%
OrthoFinder (BL+MSA)	0.0%	0.0%	0.0%	0.0%	0.0%	0.0%	0.0%	0.0%	0.0%	0.0%	0.0%	0.0%	0.0%
EggNOG	19.7%	-0.6%	11.2%	18.3%	-0.4%	11.3%	32.0%	-1.1%	28.5%	32.0%	-2.7%	25.9%	15.7%
Ensembl Compara	19.0%	5.5%	13.1%	27.1%	1.9%	18.1%	15.5%	-0.6%	13.5%	30.2%	-0.7%	24.6%	16.9%
PANTHER 8.0 (all)	2.9%	7.5%	5.1%	2.4%	6.0%	4.0%	-5.7%	-1.1%	-5.0%	16.5%	1.4%	13.3%	4.9%
phylomeDB	16.0%	-0.5%	9.0%	10.0%	-2.0%	5.3%	6.9%	-2.4%	5.6%	32.4%	-4.1%	26.2%	10.3%
**OF v best non-OF**	2.9%	-0.6%	5.1%	2.4%	-2.0%	4.0%	-5.7%	-2.4%	-5.0%	16.5%	-4.1%	13.3%	4.9%
**OF v worst non-OF**	19.7%	7.5%	13.1%	27.1%	6.0%	18.1%	32.0%	-0.6%	28.5%	32.4%	1.4%	26.2%	16.9%

Table S2a: Precision, recall and F-score for gene duplication event identification

	Flies			Primates			Metazoa			Overall		
	**Precision**	**Recall**	**F-score**	**Precision**	**Recall**	**F-score**	**Precision**	**Recall**	**F-score**	**Precision**	**Recall**	**F-score**
**OrthoFinder tree resolution**	74.3%	77.3%	75.8%	91.7%	91.5%	91.6%	60.4%	83.9%	70.3%	79.5%	85.2%	82.2%
**Overlap (no resolve)**	58.8%	65.4%	61.9%	87.7%	89.7%	88.7%	58.2%	83.3%	68.5%	71.6%	79.9%	75.5%
**DLCPpar***	91.6%	90.3%	91.0%	92.6%	92.0%	92.3%	92.5%	92.5%	92.5%	92.2%	91.5%	91.8%
**DLCpar_search***	41.8%	69.7%	52.3%	77.8%	87.5%	82.3%	64.1%	91.0%	75.2%	59.7%	81.5%	68.9%
**Forester***	19.8%	65.4%	30.4%	59.8%	89.7%	71.8%	51.7%	93.3%	66.5%	37.0%	81.4%	50.8%

Table S2b: True positives, false positives and false negatives for gene duplication event identification

	Flies			Primates			Metazoa			Overall		
	**TP**	**FP**	**FN**	**TP**	**FP**	**FN**	**TP**	**FP**	**FN**	**TP**	**FP**	**FN**
**OrthoFinder tree resolution**	9304	3221	2728	14707	1330	1359	4168	2729	799	28179	7280	4886
**Overlap (no resolve)**	7868	5502	4164	14406	2023	1660	4138	2975	829	26412	10500	6653
**DLCPpar***	10869	997	1163	14776	1173	1290	4593	375	374	30238	2545	2827
**DLCpar_search***	8388	11674	3644	14053	4013	2013	4518	2526	449	26959	18213	6106
**Forester***	7869	31905	4163	14416	9695	1650	4632	4328	335	26917	45928	6148
