# Supplementary material for: OrthoFinder: phylogenetic orthology inference for comparative genomics
Source: Genome Biol. 2019 Nov 14;20:238. doi: 10.1186/s13059-019-1832-y (PMC6857279; doi:10.1186/s13059-019-1832-y)
Supplement: Supplementary file 2 — Additional file 2. Review history. [file 13059_2019_1832_MOESM2_ESM.docx]

Review History

**First round of review**

**Reviewer 1**

**Were you able to assess all statistics in the manuscript, including the appropriateness of statistical tests used?**

There are no statistics in the manuscript.

**Have you been able to install and test the software?**

Yes

**Comments to author:**

The authors describe advances made in the OrthoFinder method. These advances address the important difficulty to infer accurate orthologs from phylogenetic gene trees. The authors have expanded the original OrthoFinder algorithm with subsequent steps of generating rooted species trees using algorithms STAG and STRIDE and rooted gene trees resolved using the new hybrid DLC analysis. This results in accurate orthologs as well as information on gene duplication events, a resolved gene tree and comparative genomics statistics. The performance of the tool has been benchmarked using the standard orthology benchmark service, and the tree resolution algorithm was compared to and evaluated against existing methods. This extended version of OrthoFinder is easy to run and the results indicate that it is a powerful, accurate, and fast tool for phylogenetic orthology inference that could benefit the field of comparative genomics. However, the claims of novelty seem exaggerated, and the claims of superior accuracy are based on results from a standard benchmark, where the raw results do not support this but have been recalculated to do so. The validity of this procedure is in question. The fact that presented accuracy results do not correspond to online results, and that only a subsection of other methods are presented raises questions about whether the results were groomed to make Orthofinder look better than it really is.

Major issues:

1. Orthofinder claims to be the first automated method for phylogenetic ortholog inference. Page2.44: "Although an automated method for phylogenetic ortholog inference does not exist,.." - but isn't PhylomeDB an automated pipeline for phylogenetic ortholog inference? It has existed for over 10 years.

2. The rest of the same sentence: ".., curated databases of orthologs obtained from analysis of phylogenetic trees have been constructed [17-20]. These curated databases generally have higher ortholog detection accuracy than score-based approximate methods [1]," in not supported by ref 1. On the contrary, ref 1 explicitly states "there is no obvious systematic difference in performance between tree-based (Ensembl, PANTHER and PhylomeDB) and graph-based methods (the rest)". The authors need to back up this claim or remove it.

3. "The full set of benchmarks, the input files and the ortholog inference results can be seen online at http://orthology.benchmarkservice.org/ and are shown in Fig. 4A-L of the main paper.". The online results are very different from Fig. 4A-L. In A-D, an entirely new measure, the pseudo F-score is introduced, and In E-H both axes are modified from the online version to "1-normalised RF distance" vs "percentage of randomly selected genes with predicted orthologs in a predefined set of species.". It is unclear how this is done precisely, and what the motivation is. It is stated in Methods that "Note that OrthoFinder can be penalized by an increased RF distance in these tests in cases where OrthoFinder judges it more parsimonious that genes are orthologs under the duplication-loss-coalescent model even if the gene tree conflicts with the species tree.", but this will affect many methods and is not really a reason for recalculating the results. It is well known that the benchmark is not perfect, but on average it does give a reasonable estimate of recall and precision. It has been approved by the orthology community, and any change from the accepted procedure runs the risk of being tuned to a specific method. The whole point with having a standard benchmark is to compare methods in standard ways, and if every new method applies their own method then this is no longer the case. At the very least, the standard benchmarking results from the the online service should be included.

4. It is unclear how public RF distances are used/recalculated. They don't correspond to the online distances in Fig 4, Supplementary table 1 and Supplementary figure 1. Are they normalised - how and why? In Figure S1A, all shown methods except for OrthoFinder have too low 1-RF distance compared to publicly available benchmark results. For these, the 1-RF distance should be increased by 0.1. In Figure S1B, OMA pairs and SonicParanoid (fast) have too low 1-RF distance, they both have an RF-distance around 0.06 in orthology benchmark server. Further, It is unclear if the pseudo F-score calculations have been based on these numbers or the correct ones. Why are only 7 methods listed in Table S1? Shouldn't fig S1D be the same as fig 4L? It is not.

5. The benchmarking of the tool seems to have been made using the QFO 2011 reference proteome dataset, and not the most recent, QFO 2018. It would be nice to have a motivation on the reasons for this, and a clarification that it is the 2011 dataset that is being used, since it is not stated in the manuscript.

6. The results from the orthology benchmark service are compared to other methods with publicly available results, but some of the tools with results available at the time of the upload of the OrthoFinder results have been excluded from the comparisons. For example OMA GETHOGS and OMA groups are excluded while some variants of the same tool are included, eg. SonicParanoid fast, default, and sensitive. It would be nice to have a motivation to why some of the methods have been excluded from the comparisons.

7. There is no definition included of what an "orthogroup" is exactly. Assuming it is the group of orthologs and inparalogs descended from a single gene in the last common ancestor of all the species being considered, why does InParanoid not have orthogroup checked in Fig. 4N?

Minor issues:

1. Line 1 page 19: 64 reference species should be 66.

2. Line 52 page 9: Should refer to Figure 2 instead of Figure 1.

3. Supplementary figure 2: Fraction of ortholog set successes: x-axis numbers should divided by 100, eg. should be 0.14 instead of 14

5. Figure 2: DenbroBLAST should be DendroBLAST

**Reviewer 2**

**Were you able to assess all statistics in the manuscript, including the appropriateness of statistical tests used?**

**Have you been able to install and test the software?**

Yes

**Comments to author:**

This is an exemplary manuscript describing a very important extension to the OrthoFinder toolkit. The authors have integfrated a series of new metrics and algorithms (largely described elsewhere) to permit unsupervised analysis of orthologue inference on very large input data, and have implemented what seem to be very robust methods for the inference of rooting on gene trees and the inference of rooted species trees given the simple input of fasta protein files from the target taxa. It installed and functioned, rapidly, out of the box.

Criticisms are minor: Figure 1 rehashes the very well known issue of best blast hit orthology inference in the face of gene duplication in a subset of analysed taxa. It does not need to be rehearsed here, and the point has been made many times previously. I suggest it is dropped, or if the authors are fond of it, moved to a supplemental figure.

Typos are similarly minimal:

p14 line 19 orthogroup twice

p17 line 17 and should be an

**Reviewer 3**

**Were you able to assess all statistics in the manuscript, including the appropriateness of statistical tests used?**

Yes, and I have assessed the statistics in my report.

**Have you been able to install and test the software?**

Yes

**Comments to author:**

Emms & Kelly present the latest advances to their previously published orthology delineation method, OrthoFinder. The main advance over the previous version of OrthoFinder is that from delineating orthogroups (lists of all genes from all species belonging to an orthologous group, as delineated by the OrthoFinder v1 implementation), substantial post-processing in a phylogeny-aware procedure additionally delineates orthologues (lists of orthologues between species pairs). The steps undertaken to achieve this produce additional useful results including a rooted species tree, rooted, resolved, and duplication-annotated gene trees, and additional comparative genomics statistics for the analysed dataset. Considerable effort has been put into integrating all the analyses under the OrthoFinder framework along with algorithmic development to be able to apply tree-based orthology approaches that are scalable to large datasets, without long runtimes.

I did not have much time for this review, but I did manage to install and run OrthoFinder v2.3.3 with the basic options and dependencies. So on the 'is this tool runnable?' front the answer is yes, and it was pleasantly simple to achieve.

Below are questions and comments on the manuscript where I feel some clarification is needed, page and line numbers according to the provided PDF.

P2L41-44: "Although an automated method for phylogenetic ortholog inference does not exist" -and similar statements in several places later in the manuscript- Unless I've misunderstood, "phylogenetic ortholog inference" is synonymous with what is often referred to as tree-based orthology inference. Automated methods for tree-based orthology inference do exist, essentially all gene-tree species-tree reconciliation methods. I believe that the sentiment behind this statement is more along the lines of … an easy-to-use pipeline, which is also scalable to large datasets, that integrates all steps required to ultimately delineate phylogenetic relationships between genes, has so far been lacking. E.g. Ensembl-Compara essentially does this, but it is certainly not easy-to-use, and other reconciliation methods would require users to perform all pre-processing steps themselves in order to be able to provide the input data to these methods. The middle paragraph on page 3 helps to put this statement in context (e.g. "some of the challenges listed above have already been addressed in isolation by a range of bioinformatic methods"), but the statement itself is somewhat over-simplified and gives the impression that OrthoFinder 2 fills a complete methods void.

P3L35-37: Similarly, "there was no automated method to infer a set of rooted gene trees given only gene sequences as input", sounds somewhat disingenuous. Notung can root or reroot gene trees, probably some other methods too, albeit yes you have to give it trees not gene sequences.

P4L54-57: "OrthoFinder can automatically infer multiple sequence alignments and phylogenetic trees using any user preferred multiple sequence alignment and tree inference method." The use of 'any' here is very bold. Reading the documentation it does indeed seem that the implementation should be able to cope with substituting many alternative tools for these steps. However, it is probably wise to remain conservative on this point as there are quite possibly some incompatible tools out there.

P6L1-10: please check that the references to figure panels are correct here, I found this rather confusing, e.g. should 4D be 4H?

P6L17-28: "All versions of OrthoFinder, irrespective of algorithmic options, had a higher average F-score/pseudo F-score across the four tests than any of the databases (Supplementary Fig. 2)." As the values are percentages from averages across all 4 test sets this obscures the SwissTree results where PANTHER performs better. But please check, Supfig. 2 does not seem to match data in SupTable1, e.g. according to the table F-scores for SwissTree range from 0.186 to 0.275 while panel A (labelled SwissTree) in the figure they are all much higher - has something been switched around here?

P7: STAG/STRIDE, would I be correct to assume that if one is working with very closely related species, or within the full set of species there is a clade of very closely-related species, that the ability to accurately infer the species tree with STAG and root it with STRIDE might be compromised due to low numbers of informative duplications? If so, how might this impact on OrthoFinder's subsequent steps?

P17L30: should the reference to Fig. 5B in fact be to Fig. 5A?

P18L46: "This showed it to be the most accurate method tested [10]." If I understand correctly this statement is based on results shown in Figure 3 of ref. 10 (OrthoFinder v1 paper). For clarity it should probably be noted that the measure assessing accuracy here is the F-score, and that individually for recall and precision other methods perform better.

P19L1-3: Which version of the reference proteomes was used? Looking online at the benchmarking service website it would appear that the 2011_04 sets were used but I could not find where this is stated in the manuscript. According to the Benchmarking paper this dataset should contain 66 species, but here 64? Figure 4 legend mentions 66 species?

P19L58-60: Browsing the results at https://orthology.benchmarkservice.org left me rather confused as to exactly how they correspond to those shown in Fig. 4A-L. SwissTree values seem to match, as do those for TreeFam-A. For Species Tree Discordance, 1-RF values seem to match the RF values, but for Generalised Species Tree Discordance almost all RF values are greater than 0.2 while the 1-RF values (as zoomed in for panel L) are almost all greater than 0.8 - I find this confusing, or perhaps I'm looking at the wrong dataset? In panel K Inparanoid (0.142) has ortholog recovery just lower than OrthoFinder (0.143) but online it sits between OrthoFinder DI and OrthoFinder BL.

Figure 2: Although some panels are described in the main text, the legend should explain all panels - currently only (g) and (h) are mentioned in the figure legend. Also, the node labels on the tree in panel (d) might be better as numbers or something else so as to avoid confusion with the panel labels themselves. DendroBLAST is misspelled.

Figure 4: Is there a reason behind the sort-order in panels A, B, C, and D? It seems possibly more logical to sort by the scores to make it easy to see the ranking of each method for each metric.

In general, is it necessary to take what seems to be a rather confrontational stance throughout the manuscript? If the points of potential confusion mentioned above are cleared up then surely the data will speak for themselves.

Typos / minor points

Throughout: ortholog and orthologue are used interchangeably in the texts and in the figures, please revise for consistency throughout the manuscript.

P1L59: that attempt identify => that attempt to identify

P3L26: [8-10, 12, 22]and => [8-10, 12, 22] and

P5L51: F-score than all version of all other => F-score than all versions of all other

P6L12: methods than can be => methods that can be

P7L10: phylogenetic relationship => phylogenetic relationships

P10L32: super-linearly in the number => super-linearly with the number

P12L12: Specie Tree Root Inference => Species Tree Root Inference

P12L19: set of unrooted orthogroup orthogroup => set of unrooted orthogroup

P15L36: However, in species-overlap method => However, in the species-overlap method

P16L35: below each child nodes => below each child node

P24L30: MSA = multiple sequences alignment => MSA = multiple sequence alignment

P24L42: the samples species => the sampled species

**Authors Response**

**Point-by-point responses to the reviewers’ comments:**

Reviewer #1

Comment 1

The authors describe advances made in the OrthoFinder method. These advances address the important difficulty to infer accurate orthologs from phylogenetic gene trees. The authors have expanded the original OrthoFinder algorithm with subsequent steps of generating rooted species trees using algorithms STAG and STRIDE and rooted gene trees resolved using the new hybrid DLC analysis. This results in accurate orthologs as well as information on gene duplication events, a resolved gene tree and comparative genomics statistics. The performance of the tool has been benchmarked using the standard orthology benchmark service, and the tree resolution algorithm was compared to and evaluated against existing methods. This extended version of OrthoFinder is easy to run and the results indicate that it is a powerful, accurate, and fast tool for phylogenetic orthology inference that could benefit the field of comparative genomics. However, the claims of novelty seem exaggerated, and the claims of superior accuracy are based on results from a standard benchmark, where the raw results do not support this but have been recalculated to do so. The validity of this procedure is in question. The fact that presented accuracy results do not correspond to online results, and that only a subsection of other methods are presented raises questions about whether the results were groomed to make Orthofinder look better than it really is.

*Response 1*

*We thank the reviewer for their supportive comments. All of the issues concerning the interpretation and presentation of the results have been addressed in the revised manuscript. We have also provided the missing competitor methods.*

Major issues

Comment 1

1. Orthofinder claims to be the first automated method for phylogenetic ortholog inference. Page2.44: "Although an automated method for phylogenetic ortholog inference does not exist,.." - but isn't PhylomeDB an automated pipeline for phylogenetic ortholog inference? It has existed for over 10 years.

*Response 1*

*We apologise for any confusion caused here. The reviewer is correct there are other pipelines that have been generated, and these underpin popular databases such as PhylomeDB, Ensembl-Compara, EggNOG and TreeFam. However, it is important to note that users cannot run these pipelines. Instead the output of these pipelines is provided as a static database by the developers of the pipelines. OrthoFinder is the first method that allows any user to run such analyses easily, quickly and with a single command. We have revised the text to make this distinction more clear.*

Comment 2

The rest of the same sentence: ".., curated databases of orthologs obtained from analysis of phylogenetic trees have been constructed [17-20]. These curated databases generally have higher ortholog detection accuracy than score-based approximate methods [1]," in not supported by ref 1. On the contrary, ref 1 explicitly states "there is no obvious systematic difference in performance between tree-based (Ensembl, PANTHER and PhylomeDB) and graph-based methods (the rest)". The authors need to back up this claim or remove it.

*Response2*

*We thank the reviewer for spotting this error. We have altered the text to correct the mistake.*

Comment 3A

"The full set of benchmarks, the input files and the ortholog inference results can be seen online at http://orthology.benchmarkservice.org/ and are shown in Fig. 4A-L of the main paper.". The online results are very different from Fig. 4A-L. In A-D, an entirely new measure, the pseudo F-score is introduced

*Response 3B*

*In the statistical analysis of classification data, the F-score (also F1-score or F-measure) is a universally applicable statistical method to measure accuracy. It considers both the precision and the recall of the test to compute its overall accuracy. The F-score is used in Figs. 4A-B in addition to the precision and recall (both obtained from the benchmark website) which are shown in Figs. 4E-F & I-J.*

*The point of using the F-score is that arbitrarily high precision/recall can be obtained at the cost of low recall/precision respectively. The F-score allows the reader to assess how well different methods manage the trade-off between the two measures. This is a key consideration for all prediction methods, and its absence on the benchmarking website is no excuse for excluding it from the manuscript. In the case of Figure 4C and D it is not possible to calculate a true F-score and so the Pseudo F-score is introduced. This is analogous to a true F-score and its calculation is described in detail the methods. Like the True F-score in Figure 4A and 4B, the pseudo F-score allows the user to directly see the trade-off between precision and recall of the different methods. The component precision and recall scores are provided Figures 4G-H & K-L*

Comment 3B

…and In E-H both axes are modified from the online version to "1-normalised RF distance" vs "percentage of randomly selected genes with predicted orthologs in a predefined set of species.". It is unclear how this is done precisely, and what the motivation is.

*Response 3C*

*The reviewer has made a small error here, Figs 4E-F are exactly as on the website. However, the reviewer is correct that Figs. 4G-H have two changes. First, the y-axis was flipped (“1-RF” instead of “RF”) so that higher ‘accuracy’ is towards the top of the graph. This was done so that the direction of “more accurate” is the same as with Figs. 4E-F (rather than opposite). Second, the x-axis is expressed as a fraction between 0-1 rather than an absolute number of completed samples between 0-50000. Again, this was also done to make these plots more consistent with the first two parts of this figure (Figs. 4E-F), and more consistent with standard practice for reporting statistical data. Overall, these changes enhance the consistency and interpretability of the test results so that a reader unfamiliar with these benchmarks, or these scores, is able to interpret them more easily.*

*Furthermore, text labels on the axes of our figures use standard statistical terminology to be as clear as possible for the reader what the axes represent. Specifically, we use the standard statistical term “precision” and “recall” instead of the less commonly used term “pos. predictive value rate” and “true positive rate”. In the same vein, we felt that for a reader without inside knowledge of the detailed steps of the benchmarking pipeline that “fraction ortholog set successes” was more understandable than “# completed tree samplings (of 50k trials)”.*

*We have added a paragraph to the methods section to explain more clearly these labels, although we stress again that the only change to the numbers is the inversion of the y-axis (right two panels) to achieve consistency (with left two panels) and the expressing of numbers as fractions, again for increased consistency.*

Comment 3C

It is stated in Methods that "Note that OrthoFinder can be penalized by an increased RF distance in these tests in cases where OrthoFinder judges it more parsimonious that genes are orthologs under the duplication-loss-coalescent model even if the gene tree conflicts with the species tree.", but this will affect many methods and is not really a reason for recalculating the results. It is well known that the benchmark is not perfect, but on average it does give a reasonable estimate of recall and precision.

*Response 3C*

*We apologise for this error. The reviewer is correct. We have now removed that paragraph.*

Comment 4

[The benchmarks have] been approved by the orthology community, and any change from the accepted procedure runs the risk of being tuned to a specific method. The whole point with having a standard benchmark is to compare methods in standard ways, and if every new method applies their own method then this is no longer the case. At the very least, the standard benchmarking results from the online service should be included.

It is unclear how public RF distances are used/recalculated. They don't correspond to the online distances in Fig 4, Supplementary table 1 and Supplementary figure 1. Are they normalised - how and why? In Figure S1A, all shown methods except for OrthoFinder have too low 1-RF distance compared to publicly available benchmark results. For these, the 1-RF distance should be increased by 0.1. In Figure S1B, OMA pairs and SonicParanoid (fast) have too low 1-RF distance, they both have an RF-distance around 0.06 in orthology benchmark server. Further, It is unclear if the pseudo F-score calculations have been based on these numbers or the correct ones. Why are only 7 methods listed in Table S1? Shouldn't fig S1D be the same as fig 4L? It is not.

*Response 4*

*We apologise for any confusion caused here. To be clear the benchmarks have not been recalculated, we have simply integrated the data from multiple independent tests into a smaller number of figures so that the data can be more easily presented in the paper. We apologise that we did not describe this very well in the original manuscript. We have now described this fully in the Methods section and also noted it in the legend to Figure 4.*

*Specifically, the species tree discordance test (STDT) figure (Fig. 4G) is the total fraction of completed tree samples over the 2 STDT plotted against their average RF score from the benchmarking website. Similarly, the generalised species tree discordance test (GSTDT) figure (Fig. 4H) is the total fraction of successes and the average RF score from the 4 GSTDT on the website. We have now stated this in the legend to Fig 4 and explained this in the section “Ortholog Benchmarking”. We note that we had already provided the four individual GSTDT results in Supplementary Fig. 1. However, we realise now that we did not make this as clear as we could. We have now added additional references to this data in the revised figure legend and at other locations in the main text. For completeness the two individual STDTs are now also included in Supplementary Figure 1 so that the complete set of phylogeny-based websites are now presented.*

*For space reasons, and for equal weighting of the four high-level tests provided by the benchmark (SwissTree, TreeFam-A, STDT & GSTDT), it was felt that this was a reasonable compromise to make. Without averaging over the STDT & GSTDT there would be double the number of plots. We wish to note that the OrthoFinder manuscript shows far more of the benchmarks than any other recently published method. For example, the SonicParanoid paper shows just one selected STDT plot in the main paper [2] and the most recent OMA paper with benchmarking in showed just two [3]. We felt that such “cherry picking” would be incorrect, but by integrating the data from all of the STDT and GSTDT results we could show the data from all 8 of the phylogeny-based benchmarks (2xSTDT, 4xGSTDT, SwissTree & TreeFAM). Moreover, we had included the 4 individual GSTDT in Supplementary Figure 1 and have now also added the 2 individual STDT. Thus the results of every bench mark test are provided in the manuscript.*

*We apologise for the discrepancy in the 1-RF distances in Supplementary Figure 1. The reviewer correctly spotted that some of the markers in Supplementary Figure 1A are incorrect and also OMA pairs and SonicParanoid (fast) in Fig. S1B too. Thank you very much for spotting this. The error occurred then the axes were flipped in the y-direction so that higher accuracy was at the top rather than the bottom. These points were accidentally left unselected and remained in place. The figure has now been regenerated from scratch. All other figures have also been checked and we have confirmed that no other figure has been affected. Thus, the pseudo F-score has been calculated from the correct numbers. The error in Fig S1A-B was a graphical error in the preparation of the figure and so did not affect the downstream numbers in any way.*

*The benchmark results from all 15 methods from Sup Fig 1 are shown in in the first tab in Sup Table S1. The spreadsheet has an additional, second tab, which compares against the database methods. Apologies if this caused confusion.*

*Fig. 4L is not be the same as Fig. S1D. Again, we apologise for any confusion here. Fig 4L is the average over all the GSTDTs as described in Response 3C. Whereas, Fig S1D is the GSTDT on just the LUCA test. This was not explained clearly in the original manuscript and we have now corrected this.*

Comment 5

The benchmarking of the tool seems to have been made using the QFO 2011 reference proteome dataset, and not the most recent, QFO 2018. It would be nice to have a motivation on the reasons for this, and a clarification that it is the 2011 dataset that is being used, since it is not stated in the manuscript.

*Response 5*

*The motivation is there were far more methods on the 2011 benchmarks (and still are as of 12th Aug 2019) and so this set provided a far more informative comparison for the paper. We agree that this should have been clarified in the manuscript, which we have now done and have highlighted the text changes for this.*

Comment 6

The results from the orthology benchmark service are compared to other methods with publicly available results, but some of the tools with results available at the time of the upload of the OrthoFinder results have been excluded from the comparisons. For example, OMA GETHOGS and OMA groups are excluded while some variants of the same tool are included, e.g. SonicParanoid fast, default, and sensitive. It would be nice to have a motivation to why some of the methods have been excluded from the comparisons.

*Response 7*

*These were not included in the original manuscript as they were different results files from the same software not specifically targeted at identifying all/only orthologs and hence scored less well than OMA pairs. (GETHOGs produces orthogroups containing orthologs and paralogs, OMA groups are a subset of ortholog relationships such that all genes in the group must be orthologs of all other genes). It was probably incorrect of us to make the judgement not to include them originally since the method’s authors had, after all, submitted them to the benchmarking server. We have now updated the manuscript to include these methods. Please note, these additional methods in general perform worse than other submissions from the same authors.*

Comment 7

There is no definition included of what an "orthogroup" is exactly. Assuming it is the group of orthologs and inparalogs descended from a single gene in the last common ancestor of all the species being considered, why does InParanoid not have orthogroup checked in Fig. 4N?

*Response 7*

*We have updated the figure text to “Multi-species orthogroup” and included a definition in the Figure Legend. InParanoid is a method for pairs of species. We accept the Reviewer’s point that technically the “orthologs + in-paralogs” provided by InParanoid constitute an orthogroup restricted to the special of just two species. However, we had the general definition of orthogroup in mind. InParanoid cannot provide orthogroups for 3 or more species, this functionality is instead provided by Hieranoid. (Multi-species) orthogroups are a means of carrying out comparative genomics across more than two species, and this functionality, which is heavily used, is what we were attempting to describe in the figure. The change to the figure text should now highlight this and avoid the technical error identified by the reviewer.*

Minor issues:

1. Line 1 page 19: 64 reference species should be 66.

2. Line 52 page 9: Should refer to Figure 2 instead of Figure 1.

3. Supplementary figure 2: Fraction of ortholog set successes: x-axis numbers should divided by 100, eg. should be 0.14 instead of 14

5. Figure 2: DenbroBLAST should be DendroBLAST

*Response to minor issues*

*Thank you for pointing these out, they have now all been corrected in the manuscript.*

Reviewer #2

This is an exemplary manuscript describing a very important extension to the OrthoFinder toolkit. The authors have integfrated a series of new metrics and algorithms (largely described elsewhere) to permit unsupervised analysis of orthologue inference on very large input data, and have implemented what seem to be very robust methods for the inference of rooting on gene trees and the inference of rooted species trees given the simple input of fasta protein files from the target taxa. It installed and functioned, rapidly, out of the box.

*Response*

*We thank the Reviewer for their supportive comments.*

Comment 1

Criticisms are minor: Figure 1 rehashes the very well known issue of best blast hit orthology inference in the face of gene duplication in a subset of analysed taxa. It does not need to be rehearsed here, and the point has been made many times previously. I suggest it is dropped, or if the authors are fond of it, moved to a supplemental figure.

*Response 2*

*We very much appreciate the Reviewers concern here. The Reviewer is correct, this issue is well-known in the ortholog inference community. However, this is not a well-known issue in the biological sciences community whose research is dependent on accurate orthology inference. As this manuscript is aimed at the wider community, we strongly feel that this figure is an essential part of justifying the motivation for use of a phylogenetic approach to ortholog inference. We hope that by including it, we will raise awareness of the problem to a more general audience and help people understand why orthology inference can be challenging.*

Typos are similarly minimal:

p14 line 19 orthogroup twice

p17 line 17 and should be an

*Response*

*Thank you for identifying these, they have been corrected.*

Reviewer #3

Emms & Kelly present the latest advances to their previously published orthology delineation method, OrthoFinder. The main advance over the previous version of OrthoFinder is that from delineating orthogroups (lists of all genes from all species belonging to an orthologous group, as delineated by the OrthoFinder v1 implementation), substantial post-processing in a phylogeny-aware procedure additionally delineates orthologues (lists of orthologues between species pairs). The steps undertaken to achieve this produce additional useful results including a rooted species tree, rooted, resolved, and duplication-annotated gene trees, and additional comparative genomics statistics for the analysed dataset. Considerable effort has been put into integrating all the analyses under the OrthoFinder framework along with algorithmic development to be able to apply tree-based orthology approaches that are scalable to large datasets, without long runtimes.

I did not have much time for this review, but I did manage to install and run OrthoFinder v2.3.3 with the basic options and dependencies. So on the 'is this tool runnable?' front the answer is yes, and it was pleasantly simple to achieve.

Below are questions and comments on the manuscript where I feel some clarification is needed, page and line numbers according to the provided PDF.

*Response*

*We thank the Reviewer for the supportive comments and have addressed each of the issues raised below.*

Comment 1

P2L41-44: "Although an automated method for phylogenetic ortholog inference does not exist" -and similar statements in several places later in the manuscript- Unless I've misunderstood, "phylogenetic ortholog inference" is synonymous with what is often referred to as tree-based orthology inference. Automated methods for tree-based orthology inference do exist, essentially all gene-tree species-tree reconciliation methods. I believe that the sentiment behind this statement is more along the lines of … an easy-to-use pipeline, which is also scalable to large datasets, that integrates all steps required to ultimately delineate phylogenetic relationships between genes, has so far been lacking. E.g. Ensembl-Compara essentially does this, but it is certainly not easy-to-use, and other reconciliation methods would require users to perform all pre-processing steps themselves in order to be able to provide the input data to these methods. The middle paragraph on page 3 helps to put this statement in context (e.g. "some of the challenges listed above have already been addressed in isolation by a range of bioinformatic methods"), but the statement itself is somewhat over-simplified and gives the impression that OrthoFinder 2 fills a complete methods void.

*Response 1*

*We thank the Reviewer for this advice. In retrospect we fully accept that P2L41-44 was poorly qualified. Our intention had been to refer to a software tool for species-wide ortholog inference from proteomes as is implemented in InParanoid or OMA or similar tools. To address this issue, we have i) Added text to the introduction discussing Ensembl-Compara and other similar pipelines used for phylogenetic databases ii) Removed claims that other methods do not exist iii) Referred more specifically to our method as an automated software tool for ortholog inference from proteomes.*

*The changes to the text have been highlighted in the manuscript. In the statements highlighted by the Reviewer we hadn’t intended to refer to the individual steps that have to be performed, e.g. reconciliation given an already calculated gene tree, but instead wished to discuss the entire process. We believe the changes to the manuscript now clarify this.*

Comment 2

P3L35-37: Similarly, "there was no automated method to infer a set of rooted gene trees given only gene sequences as input", sounds somewhat disingenuous. Notung can root or reroot gene trees, probably some other methods too, albeit yes you have to give it trees not gene sequences.

*Response 2*

*We thank the Reviewer for this comment. This was an incorrect choice of terminology on our part, what we wished to refer to was, “the inference of a complete set of rooted gene trees from a set of species proteomes”, and referring to “gene sequences as input” did not specify this. We have updated the text to be more specific. Additionally, we now no longer claim that there is no automated method for this.*

Comment 3

P4L54-57: "OrthoFinder can automatically infer multiple sequence alignments and phylogenetic trees using any user preferred multiple sequence alignment and tree inference method." The use of 'any' here is very bold. Reading the documentation it does indeed seem that the implementation should be able to cope with substituting many alternative tools for these steps. However, it is probably wise to remain conservative on this point as there are quite possibly some incompatible tools out there.

*Response 3*

*Point accepted, ‘any’ is possibly too bold and we have changed it to “most”. The reason for making this statement is that I think it would even be very difficult to specifically design a tool with the intention of making it not work with OrthoFinder. Any incompatibility e.g. non-matching file types can be resolved by a wrapper script that first calls a file conversion program and then calls the method on the converted file. Similarly, a wrapper can be used with a program that uses an interactive prompt. The OrthoFinder mechanism has been designed to have the required flexibility to deal with any such problems.*

Comment 4

P6L1-10: please check that the references to figure panels are correct here, I found this rather confusing, e.g. should 4D be 4H?

*Response 4*

*We thank the Reviewer for spotting this error. Also the last two references to 4C should have been 4H & G and these have also now been updated.*

Comment 5

P6L17-28: "All versions of OrthoFinder, irrespective of algorithmic options, had a higher average F-score/pseudo F-score across the four tests than any of the databases (Supplementary Fig. 2)." As the values are percentages from averages across all 4 test sets this obscures the SwissTree results where PANTHER performs better.

*Response 5*

*The Reviewer is correct. We have updated the manuscript text to read, “All versions of OrthoFinder, irrespective of algorithmic options, had a higher F-score/pseudo F-score across each of the four tests than any of the databases with the one exception of PANTHER on the Species Tree Discordance Test”.*

Comment 6

But please check, Supfig. 2 does not seem to match data in SupTable1, e.g. according to the table F-scores for SwissTree range from 0.186 to 0.275 while panel A (labelled SwissTree) in the figure they are all much higher - has something been switched around here?

*Response 6*

*We thank the Reviewer for spotting this mistake. The values for SwissTree/TreeFam-A and those for STDT/GSTDT had been switched, this has now been corrected.*

Comment 7

P7: STAG/STRIDE, would I be correct to assume that if one is working with very closely related species, or within the full set of species there is a clade of very closely-related species, that the ability to accurately infer the species tree with STAG and root it with STRIDE might be compromised due to low numbers of informative duplications? If so, how might this impact on OrthoFinder's subsquent steps?

*Response 7*

*For the (unrooted) species tree inference, STAG would not have any problems in this case of few duplications. It is designed to work even if there are lots of duplications but in no way requires any duplications in order to infer the tree.*

*However, with very closely related species there might not be enough informative duplications to root the species tree accurately with STRIDE. If it were just a sub-clade within the species tree of very closely-related species this would not be a problem. The duplications elsewhere in the tree provide all the information that is required. The advantage of the STRIDE approach for rooting the species tree is that having the correct root for the species tree only affects ortholog inference if there have been duplications [Ref. 1: Fig 1]. If there are few duplications then it may not be possible to accurately locate the root of the species tree. However, the corollary of this is that if there are very few duplications there will be very few genes for which ortholog inference is impacted by the incorrect root. If there are duplications in just a subset of the species then the STRIDE method will be able to exclude the root from that clade (ensuring accurate ortholog inference within that clade since the direction of time will be correct throughout the clade) but STRIDE will not be able to pinpoint where in the remaining tree the root is (but this will have little impact on ortholog inference since there are few duplications outside of the duplication-rich clade).*

*A description of these considerations was removed from the final draft of the manuscript as it seemed too technical but we have now reinserted it in the Methods section, “Species tree inference and rooting” since this does add to the paper.*

Comment 8

P17L30: should the reference to Fig. 5B in fact be to Fig. 5A?

*Response 8*

*Corrected, thank you.*

Comment 9

P18L46: "This showed it to be the most accurate method tested [10]." If I understand correctly this statement is based on results shown in Figure 3 of ref. 10 (OrthoFinder v1 paper). For clarity it should probably be noted that the measure assessing accuracy here is the F-score, and that individually for recall and precision other methods perform better.

*Response 9*

*We have now added this.*

Comment 10

P19L1-3: Which version of the reference proteomes was used? Looking online at the benchmarking service website it would appear that the 2011_04 sets were used but I could not find where this is stated in the manuscript. According to the Benchmarking paper this dataset should contain 66 species, but here 64? Figure 4 legend mentions 66 species?

*Response 10*

*The 2011_04 version was the version with the most methods compared and so this was used throughout the manuscript. We have added this information to the manuscript in the relevant locations, as highlighted. 64 has now been to corrected 66 at P19L1-3.*

Comment 11

P19L58-60: Browsing the results at https://orthology.benchmarkservice.org left me rather confused as to exactly how they correspond to those shown in Fig. 4A-L. SwissTree values seem to match, as do those for TreeFam-A. For Species Tree Discordance, 1-RF values seem to match the RF values, but for Generalised Species Tree Discordance almost all RF values are greater than 0.2 while the 1-RF values (as zoomed in for panel L) are almost all greater than 0.8 - I find this confusing, or perhaps I'm looking at the wrong dataset? In panel K Inparanoid (0.142) has ortholog recovery just lower than OrthoFinder (0.143) but online it sits between OrthoFinder DI and OrthoFinder BL.

*Response 11*

*The numbers the Reviewer has seen online for the Generalised Species Tree Discordance (GSTD) and the Species Tree Discordance (STD) Test are for just one of the tests for each of these. There are in fact four tabs for four tests on https://orthology.benchmarkservice.org for GSTD and two for STD. The figures in the paper summarise these by presenting the total fraction of successes across the four/two tests and the average normalised RF distance across these successes for the GSTD and STD tests respectively. A paragraph has been added to the “Orthology Benchmarking” section to describe this. Additionally, the four individual GSTD tests were included in the original manuscript as Supplementary Figure 1. We felt this provided the best mix of summarising the complete set of orthology benchmarks in the main paper while breaking them down into component parts in the Supplementary Figures.*

Comment 12

Figure 2: Although some panels are described in the main text, the legend should explain all panels - currently only (g) and (h) are mentioned in the figure legend. Also, the node labels on the tree in panel (d) might be better as numbers or something else so as to avoid confusion with the panel labels themselves. DendroBLAST is misspelled.

*Response 12*

*We apologise for this error (also noted in the response to comment 4 above). All the panels are now described in the legend and referenced in the text. The node labels in panel (d) have also been changed and the spelling mistake corrected.*

Comment 13

Figure 4: Is there a reason behind the sort-order in panels A, B, C, and D? It seems possibly more logical to sort by the scores to make it easy to see the ranking of each method for each metric.

*Response 13*

*We thank the Reviewer for this suggestion. The methods have now been reordered by ranking.*

Comment 14

In general, is it necessary to take what seems to be a rather confrontational stance throughout the manuscript? If the points of potential confusion mentioned above are cleared up then surely the data will speak for themselves.

*Response 14*

*Thank you for this comment and our apologies that the manuscript came across this way. Hopefully, the changes we have made make it come across less like this by recognising more other available methods. We have revised manuscript in response to the all the Reviewers’ comments and thank the Reviewers for their help in identifying potential points of confusion were the manuscript could be improved.*

Typos / minor points

Throughout: ortholog and orthologue are used interchangeably in the texts and in the figures, please revise for consistency throughout the manuscript.

P1L59: that attempt identify => that attempt to identify

P3L26: [8-10, 12, 22]and => [8-10, 12, 22] and

P5L51: F-score than all version of all other => F-score than all versions of all other

P6L12: methods than can be => methods that can be

P7L10: phylogenetic relationship => phylogenetic relationships

P10L32: super-linearly in the number => super-linearly with the number

P12L12: Specie Tree Root Inference => Species Tree Root Inference

P12L19: set of unrooted orthogroup orthogroup => set of unrooted orthogroup

P15L36: However, in species-overlap method => However, in the species-overlap method

P16L35: below each child nodes => below each child node

P24L30: MSA = multiple sequences alignment => MSA = multiple sequence alignment

P24L42: the samples species => the sampled species

*Response 15*

*Thank you for identifying these, they have all now been corrected.*

*Additional Notes for Reviewers*

*Notung was included in the original version of the manuscript in the tests for accuracy at identifying gene duplication events. However, its results were identical to those of Forester. The author of Notung has communicated to us that the reason for this is that Notung uses branch support values to try and improve the accuracy of its DL analysis (i.e. over that achieved by Forester) and the trees in the test set had no support values. The author of Notung has asked that we not include Notung in Figure 5, which showed it had identical accuracy to Forester, since this hides the fact that it is a more accurate method when provided with support values, and could mislead readers of this paper. We agreed to this as a comparison between Notung and Forester is not an significant point of the paper and we didn’t wish to cause confusion over these methods.*

*References*

*[1] Emms DM, Kelly S: STRIDE: Species Tree Root Inference from Gene Duplication*

*Events. Molecular Biology and Evolution 2017:msx259-msx259.*

*[2] Salvatore Cosentino and Wataru Iwasaki (2018) SonicParanoid: fast, accurate, and easy orthology inference. Bioinformatics, Volume 35, Issue 1, 1 January 2019, Pages 149–151*

*[3] Orthologous Matrix (OMA) algorithm 2.0: more robust to asymmetric evolutionary rates and more scalable hierarchical orthologous group inference. Bioinformatics, 2017, 33:14, (pp. i75–i82)*

**Second round of review**

**Reviewer 1:**

Comment 1

The revised manuscript allays most of the raised issues. It was a bit challenging to read the response letter as it has incorrectly numbered response sections, but it seems that what at first appeared to be recalculation of benchmark scores was due to poor labeling and descriptions, and most of this has been fixed. However a few issues still remain.

Comment 2

Concerning original comment 3: The provided explanation is fine, but it would be good to change the highly confusing figure text “X-axis: Total fraction of randomly selected genes with predicted orthologs in a predefined set of species ...” to “Total fraction of completed tree samplings” as stated on the source benchmark.

Comment 3

Concerning original comment 5. Although there is now a reasoning behind choosing the QFO 2011 benchmark, it is quite old and some of the methods have been update since then, for instance OrthoInspector for which the latest version is only run on the QFO 2018 benchmark. Therefore it would be prudent to include QFO 2018 benchmark results too.

Comment 4

Concerning original comment 6. There are still methods with public online results missing from figs 4 and S2, e.g. OMA 2.0 and EggNog 5.0 (groups).

**Reviewer 3:**

The authors have satisfactorily responded to my comments. The previous confusing discrepancies with the online benchmarking results have now been resolved.

**Authors Response**

**Point-by-point responses to the reviewers’ comments:**

Reviewer 1:

Comment 1

The revised manuscript allays most of the raised issues. It was a bit challenging to read the response letter as it has incorrectly numbered response sections, but it seems that what at first appeared to be recalculation of benchmark scores was due to poor labeling and descriptions, and most of this has been fixed. However a few issues still remain.

*Response 1*

*We thank the reviewer for highlighting the outstanding errors. These have now been addressed as described below.*

Comment 2

Concerning original comment 3: The provided explanation is fine, but it would be good to change the highly confusing figure text “X-axis: Total fraction of randomly selected genes with predicted orthologs in a predefined set of species ...” to “Total fraction of completed tree samplings” as stated on the source benchmark.

*Response 2*

*We have changed the text as requested.*

Comment 3

Concerning original comment 5. Although there is now a reasoning behind choosing the QFO 2011 benchmark, it is quite old and some of the methods have been update since then, for instance OrthoInspector for which the latest version is only run on the QFO 2018 benchmark. Therefore it would be prudent to include QFO 2018 benchmark results too.

*Response 3*

*We have now added the 2018 benchmarks as "Additional file 1, Figure S2" and highlighted these results in the appropriate results section. (As with the previous benchmarks all versions of OrthoFinder were more accurate than all other methods and so the new benchmarks do not change our conclusions).*

Comment 4

Concerning original comment 6. There are still methods with public online results missing from figs 4 and S2, e.g. OMA 2.0 and EggNog 5.0 (groups).

*Response 4*

*EggNOG 5.0 (groups) was not available when we'd prepared the figure, we have now updated the Figure S3 to include it. There may have been some confusion with OMA since there are five OMA methods listed on the benchmarks website, but two of these are marked as superseded and so haven't been included: "OMA GETHOGs" & "OMA Groups (RefSet5)". We can confirm that, as requested, all three versions OMA 2.0 are included in Figure 4: OMA Pairs, OMA GETHOGs & OMA Groups.*
